# Conductance Quantization in Resistive Random Access Memory

**DOI:** 10.1186/s11671-015-1118-6

**Published:** 2015-10-26

**Authors:** Yang Li, Shibing Long, Yang Liu, Chen Hu, Jiao Teng, Qi Liu, Hangbing Lv, Jordi Suñé, Ming Liu

**Affiliations:** Key Laboratory of Microelectronics Devices and Integrated Technology, Institute of Microelectronics, Chinese Academy of Sciences, Beijing, 100029 China; Lab of Nanofabrication and Novel Device Integration, Institute of Microelectronics, Chinese Academy of Sciences, Beijing, 100029 China; Department of Materials Physics and Chemistry, University of Science and Technology Beijing, Beijing, 100083 China; Departament d’Enginyeria Electrònica, Universitat Autònoma de Barcelona, Bellaterra, 08193 Spain

**Keywords:** Resistive random access memory (RRAM), Resistive switching (RS), Conductive filament (CF), Conductance quantization

## Abstract

The intrinsic scaling-down ability, simple metal-insulator-metal (MIM) sandwich structure, excellent performances, and complementary metal-oxide-semiconductor (CMOS) technology-compatible fabrication processes make resistive random access memory (RRAM) one of the most promising candidates for the next-generation memory. The RRAM device also exhibits rich electrical, thermal, magnetic, and optical effects, in close correlation with the abundant resistive switching (RS) materials, metal-oxide interface, and multiple RS mechanisms including the formation/rupture of nanoscale to atomic-sized conductive filament (CF) incorporated in RS layer. Conductance quantization effect has been observed in the atomic-sized CF in RRAM, which provides a good opportunity to deeply investigate the RS mechanism in mesoscopic dimension. In this review paper, the operating principles of RRAM are introduced first, followed by the summarization of the basic conductance quantization phenomenon in RRAM and the related RS mechanisms, device structures, and material system. Then, we discuss the theory and modeling of quantum transport in RRAM. Finally, we present the opportunities and challenges in quantized RRAM devices and our views on the future prospects.

## Review

### Introduction

The persistent perusing of massive storage volume has been driving the scaling-down process of memory devices for decades. Memories characterized by low-power consumption and low fabrication cost are needed. Predominant flash memory has met a scaling-down limitation around 10-nm magnitude [1, 2]. Therefore, intensive studies have been carried out in seeking for the next-generation memories. Resistive random access memory (RRAM) has become one of the most promising candidates for the next-generation memory [3–14] because of the intrinsic excellent scalability, simple metal-insulator-metal (MIM) structure, low fabrication cost, 3D integration feasibility, and promising performances in speed, power, endurance, retention, etc. RRAM stores information based on the resistive switching effect. Under appropriate external electrical field, the resistance state of the RRAM device can be reversibly switched between a high resistance state (HRS) or OFF-state and a low resistance state (LRS) or ON-state. There are two resistive switching modes, i.e., unipolar and bipolar switching operations under the same or opposite bias polarities, respectively, which are closely related to the different material systems and the different switching mechanisms. The resistive switching can be a uniform or localized phenomenon. Uniform switching proportionally scales with the total area of the switching material, while localized switching is usually based on the formation and disruption of conductive filament (CF).

Abundant resistive switching materials, electrode materials, and their various interfaces are involved in RRAM switching mechanisms which are rather complex. Rich electrical, thermal, magnetic, and optical effects are therefore presented. Typical physical/chemical effects accompanied in resistive switching processes and in HRS/LRS states include electrochemical/thermochemical reactions [15–27], metal-insulator transition [28, 29], magnetic modulation [30–47], etc. In this regard, the RRAM device can serve as a rich platform for studying the multiple physical/chemical effects. In the CF-type RRAM device, when the CF in the resistive switching (RS) layer is formed, RRAM changes to LRS. If the CF is ruptured, the device switches back to HRS. The formation and rupture of the CF can be understood as cation or anion migration under applied voltage companied by electrochemical reaction of the metal or oxygen vacancies. Therefore, CF is believed to be consisted of metal or oxygen vacancies. The dimension of the CF can be electrically modulated to be in the order of several tens to a few nanometers, which has been evidenced by the observation of high-resolution transmission electron microscopy (HRTEM) [20, 48–60], scanning TEM (STEM) [59], and atomic force microscopy (AFM) [61–63]. In the localized filamentary switching, the scaling down of the RRAM device [64] would not influence its memory characteristics until the area is approaching the CF magnitude. As the CF size is in the range of nanoscale to atomic size, which is comparable to the mean free path (Fermi wavelength) of conduction electron, the scattering might be absent, resulting in ballistic electron transport [65] and the quantized conductance (QC) [66–68]. In recent studies, conductance quantization phenomena have been proved to exist in the atomic-sized CF in RRAM [69–72], and the interest for studying them continues. Revealing the QC effect is of great significance to deeply understand the physics of RS mechanism in mesoscopic dimension, which is important to control the performance, reliability, and variability [73, 74] of RRAMs and to advance their practical application as non-volatile memories. At the same time, if the conductance quantization behaviors can be well modulated, it in turn can be utilized to realize the multi-level storage for ultra-high-density memory applications. Thus, summarizing and discussing the QC effect in RRAM is very necessary. In this review paper, we focus our attention on the recent development of the research on the QC effect in CF-based non-volatile RS devices including basic QC phenomenon in RRAM, RS mechanisms, device structures, materials, theory, and modeling of conductance quantization in RRAM.

### Operating Principles and RS Mechanism of RRAM

**Fig. 1:**
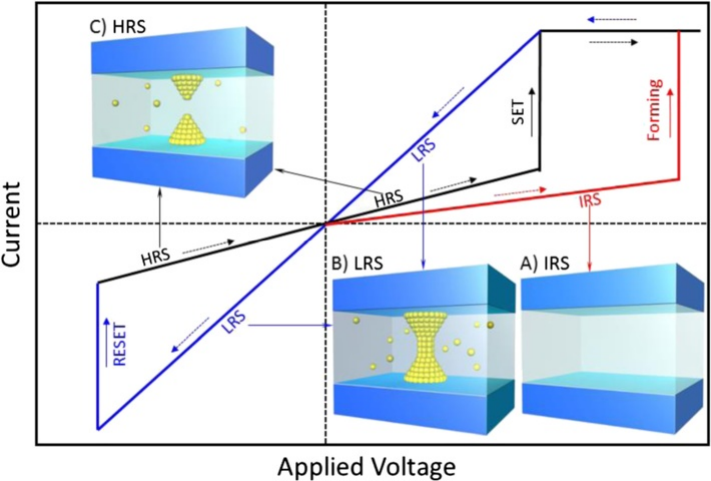
Schematic *I–V* curves of resistive switching process in a CF-type bipolar RRAM device. Insets *A*–*C* show the different resistance states of the device during the switching process. In most cases, the fresh RRAM device shows a very high initial resistance state (IRS) with few defects (inset *A*). In a positive bias sweep, when the voltage increases to a comparative high voltage (*V*_Forming_), the device switches to the low resistance state (LRS) with a conducting filament formed in the RS layer (inset *B*). Then, in a negative voltage sweep, when the voltage reaches a critical value (*V*_RESET_), the device switches from LRS to the high resistance state (HRS), corresponding to a RESET transition in which the CF is ruptured (inset *C*). At last, in another positive sweep, the device will switch to LRS again, with the filament reconnected (inset *B*). This process is called as SET, with the SET voltage (*V*_SET_) much lower than (*V*_Forming_). If the device has a good endurance, the above SET and RESET switching can be reproducibly and successively carried out for a large number of cycles

In RRAM cell with MIM structure, non-volatile data storage is achieved through the reversible resistive switching between HRS and LRS, which are utilized to store the digits “0” and “1.” RS is often based on the creation and partial destruction of CF. Dependent on the polarity of the external electrical field, RS is usually classified into two modes, unipolar and bipolar switching. The transition from HRS to LRS and that from LRS to HRS are called as the SET and RESET switching, respectively. In some cases, if the prepared RS layer in fresh cell is very insulating with low amount of defects, a forming process with high voltage is necessary to soft breakdown the RS layer to trigger the subsequent reproducible RESET and SET switching. Figure 1 shows the schematic *I–V* curves in RS and the corresponding states of CF-type RRAM device.

Compared with the prototypical non-volatile memories (NVMs) such as magnetic random access memory (MRAM) based on the giant or tunneling magnetoresistance effect [75–79] and phase change random access memory (PRAM) based on the reversible phase transitions between amorphous and crystalline states of phase change materials [80–84], RRAM, an emerging NVM, has shown various complex resistive switching mechanisms, which is closely dependent on the different types of switching layer and electrode materials and also dependent on the different operation methods [85–87]. To date, the resistive switching mechanism in RRAM has been widely accepted to be mainly attributed to the reduction/oxidation (redox) electrochemistry mechanism, which can operate in the bulk RS layer, along CFs in the RS layer, and/or at the RS layer/metal contact interfaces in the MIM structure. Redox-based RRAM [3, 15, 19, 88–95] can be further classified into two main types, “nanoionic” (including electrochemical metallization (ECM) [17–20, 90, 94, 96–101] and valence change mechanism (VCM) [5, 51, 61, 91, 102–112]) and “thermochemical mechanism (TCM)” (i.e., fuse/antifuse) [91, 113, 114]. In ECM and VCM devices, the diffusion or drift of charged species (Ag^+^ or Cu^+^ cations), O anions or oxygen vacancies (Vos) in the RS layer are driven by the ion drift/redistribution and the redox electrochemistry mechanisms under an electrostatic drift field. While in TCM devices, it is driven by a thermal gradient diffusion mechanism. In fact, in a practical RRAM device, the RS process is very complicated, with multiple mechanisms simultaneously existing, but a certain one is predominant. It is worth pointing out that TCM is sometimes confused with unipolar VCM. In some cases, the ECM device is also called as conductive bridge random access memory (CBRAM) [115–119], programmable metallization cell (PMC) [120–122], and atomic switch [115–119, 123, 124]. If TCM dominates the RS, the resistance switching is unipolar. On the contrary, if ECM or VCM is dominant, the switching is usually bipolar. ECM devices are cation migration-based RRAMs, while VCM and TCM devices can be summed up into anion migration-based RRAMs. Plenty of dielectric materials have been found to show the redox-based resistance switching effect, including perovskites, solid-state electrolytes, chalcogenides, transition metal oxides, silicon dioxide, metal nitrides, organic complexes, polymers, etc., among which HfO_2_ and TaO_x_ are most widely investigated and most competitive for the practical applications. Table 1 lists the three types of typical redox-based RS mechanisms and their corresponding material system, *I*–*V* curves, and operation polarity.

**Table 1:** The classification of redox-based resistive switching mechanisms and operation principles of redox-based RRAM

Switching mechanism	Electrochemical metallization (ECM)	Valence change mechanism (VCM)	Thermochemical mechanism (TCM)
Dominating charged species	Metal cations	O anions or oxygen vacancies (Vo)	O anions or oxygen vacancies (Vo)
Intrinsic nature of CF	Metal CF	Vo-CF (bipolar)	Vo-CF (unipolar)
Dominating driving force	External electric field	External electric field	Thermal gradient
Primary operation principle	SET process: (1) The active TE material (Ag, Cu, Ni) in the interface is oxidized under positive electric field; (2) the cations (Ag^+^, Cu^+^ or Cu^2+^, Ni^+^) drift into the RS layer; (3) the cations are reduced back from the BE/RS-layer interface or from the bulk RS layer or even from the TE/RS layer, depending on the difference between the drift velocity of cations and electrons; (4) metal CF is formed to connect BE and TE, with the reduction process continuing.	SET process: (1) Under positive electric field, TE material in the TE/RS-layer interface is oxidized and O^2−^/Vo is generated; (2) O ions drift to TE or O vacancies drift to BE through the RS layer to form Vo-CF. The valence states of corresponding cations are changed.	SET (antifuse) process: O vacancies are generated, diffused, and redistributed to form Vo-CF in the bulk RS layer under the thermal gradient induced by electric field. The valence states of corresponding cations are changed.
	RESET process: Under the opposite electric field, the metal atoms in the CF are oxidized and drift away, thus CF is partially ruptured.	RESET process: Under the opposite electric field, O ions migrate back to the bulk RS layer to recombine with O vacancies in the CF, thus CF is partially ruptured.	RESET (fuse) process: CF is ruptured or fused as a result of joule heating along the CF through the thermal diffusion process of O vacancies.
Typical RS materials	Ion-conducting solid electrolyte (sulfides, selenides, or telluride of Ge, As, Sb, or Ga) such as Ag_2_S [53], GeSe [224], Cu_2_S [172, 225], Ag_2_Se [226], Ag-Ge-Se [227], (AgI)_0.5_(AgPO_3_)_0.5_ [228], etc.; Binary or complex oxides such as HfO_2_ [215, 222, 229, 230], ZrO_2_ [231, 232], SiO_2_ [233], WO_3_ [234], TaO_x_ [235], GdO_x_ [236], etc.	Transition metal oxides (TMOs) such as TiO_2_ [51, 87], HfO_2_ [106], ZrO_2_ [112], SrTiO_3_ [5, 61], TaO_x_ [102], WO_3_ [111], etc.; doped SiO_2_ [104, 107–109]; amorphous C [103, 105, 110], etc.	Transition metal oxides (TMOs) such as HfO_2_ [69, 70, 216, 237], NiO [22, 238–244], CoO [245], CuO [246], Fe_2_O_3_ [247], etc.
Typical electrode materials	(1) Top electrode (TE): an electrochemically active metal such as Ag, Cu, and Ni.	(1) Top electrode (TE): a low work function metal not easily reduced back after oxidation, such as Ti, Al, and Nb.	(1) Top electrode (TE): inert electrodes such as Pt, Pd, Ir, Ru, W, Au, etc.
	(2) Bottom electrode (BE): an electrochemically inert counter electrode such as Pt, Pd, Ir, Ru, W, Au, etc.	(2) Bottom electrode (BE): inert electrodes, such as Pt, Pd, Ir, Ru, W, Au, etc.	(2) Bottom electrode (BE): inert electrodes such as Pt, Pd, Ir, Ru, W, Au, etc.
Dominating material	Electrode	RS layer and electrode	RS layer
Typical *I*–*V* curve	(Cu/HfO_2_/Pt)	(Ti/HfO_2_/Pt)	(Pt/HfO_2_/Pt)
Operation polarity	Bipolar	Bipolar	Unipolar
RS type	Localized	Localized	Localized

Except the redox RS mechanism, the insulator-metal transition (IMT) or Mott transition in MIM structure can also contribute to RS effect. The corresponding resistive switching device is called as Mott memory and sometimes referred as correlated electron random access memory (CeRAM). In this type of memory device, the charge injection under the external electrical field induces the transition from weakly correlated electron state to strongly correlated electron state, which is activated by a critical electron population [125–127]. Electronic switches and memory elements based on the Mott transition have been explored using several typical material systems involved in Mott memory including VO_2_ [128, 129], NiO [126, 127, 130], SrTiO_3_ [131], SmNiO_3_ [132], etc. The quasi two-dimensional electron gas (2DEG) formed at the interface between complex oxides has also been reported to show the metal-insulator transition effect [133–136]. However, in this paper, we will focus on the redox-based filamentary RS mechanisms that have been shown to support the conductance quantization effect.

### Conductance Quantization in RRAM

**Fig. 2:**
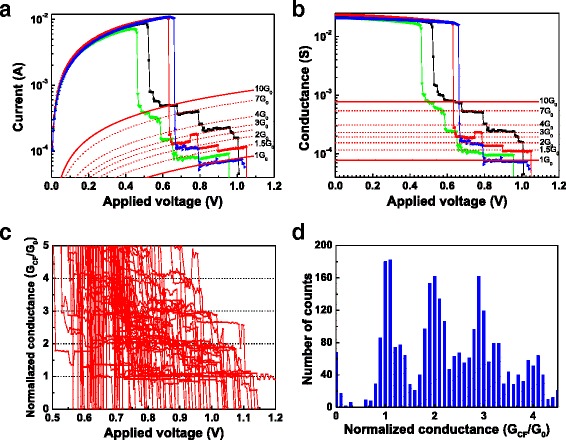
Conductance quantization phenomenon measured in the RESET process of a Pt/HfO_2_/Pt unipolar RRAM device. **a** *I* − *V* curves in four RESET cycles. **b** *G* − *V* curves corresponding to (**a**). *G* is defined as *I*/*V*. The RESET transients of the device show discrete states with half-integer multiples of the conductance quantum. The *red lines* in (**a**) and (**b**) are “guides to the eye” and correspond to *I* = *nG*_0_ *V* or *G* = *nG*_0_ with *n* = 1, 1.5, 2, 2.5, 3, 4, 7, and 10. Discrete conductance levels at about 1 *G*_0_, 1.5 *G*_0_, 2 *G*_0_, 2.5 *G*_0_, 3 *G*_0_, 4 *G*_0_, etc. are evident. **c** Evolution of CF conductance in the last stage of 100 successive RESET switching cycles of the Pt/HfO_2_/Pt device. Discrete conductance levels at 1 *G*_0_, 2 *G*_0_, 3 *G*_0_, 4 *G*_0_, etc. are revealed. **d** Histogram of normalized conductance collected at the step-like gradual RESET phase in 100 successive RESET cycles. Conductance peaks at integer and semi-integer multiples of *G*_0_ are clearly present

The size of the CF can be modulated to the range of nanoscale to atomic size in both RESET and SET process. The modulation process can be achieved through specific electrical operations, especially in the RRAM devices showing progressive RESET/SET behavior [137]. This is similar to the performance of memristor [6, 138–142]. When CF is controlled to be thin enough to show atomic size, the quantum-sized effect [69] will appear in the CF-type RRAM devices. Conductance quantization phenomenon is an important representation of quantum-sized effect. Figure 2 gives an example of conductance quantization behavior observed in RRAM. The measured current–voltage (Fig. 2a) and corresponding conductance-voltage curves (Fig. 2b) show that the conductance quantization phenomenon appears in the RESET process of a Pt/HfO_2_/Pt unipolar RRAM device. Obvious quantized conductance steps with multiples of *G*_0_ can be observed, in other words, abrupt conductance transitions of the order of *G*_0_ between well-defined discrete states can be found in the final stages of the RESET transient. *G*_0_ = 2*e*^2^/*h* is the quantum of conductance, with the value of 12.9 kΩ^−1^ or 77.5 μs, where *e* is the electron charge and *h* is Planck’s constant. Quantized conductance observed in the practical materials usually presents fluctuation with a certain degree, so statistical analysis on plenty of experimental data is often made use of to intrinsically reveal this effect. Figure 2c further shows the evolution of CF conductance of this device in the last stage of 100 successive RESET switching cycles. By collecting the conductance data at the step-like gradual RESET phase in the 100 successive RESET cycles in Fig. 2c, we can plot the histogram of normalized conductance, as shown in Fig. 2d. Conductance levels and peaks at 1 *G*_0_, 2 *G*_0_, 3 *G*_0_, 4 *G*_0_, etc. are clearly displayed in Fig. 2c, d, respectively.

**Fig. 3:**
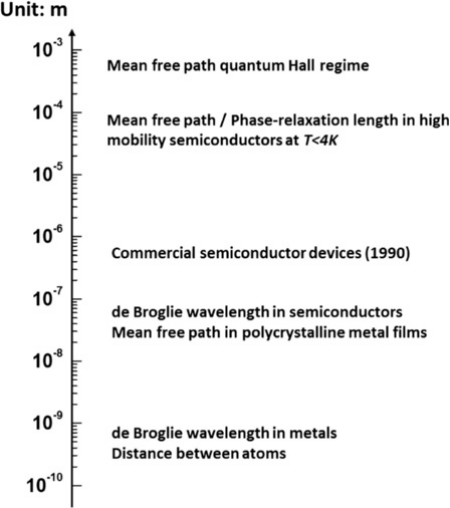
Three characteristic length scales related to quantum conductance phenomenon. The three characteristic length scales are the following: (1) the de Broglie wavelength, which is related to the kinetic energy of the electrons; (2) the mean free path, which is the distance that an electron travels before its initial momentum is destroyed; and (3) the phase-relaxation length, which is the distance that an electron travels widely from one material to another and is also strongly affected by temperature, magnetic field, etc. A conductor will show conductance quantization behavior if any of its three dimensions is smaller than the three characteristic length scales mentioned above. Reproduced with permission [143]

Only the size of a conductor is small enough will the quantized conductance effect appear. In fact, as pointed out by Datta, if any of the three dimensions of a conductor is smaller than one of the three characteristic length scales [143]: (1) de Broglie wavelength of electrons; (2) the mean free path of electrons; and (3) the phase-relaxation length of electrons, the conductor will show conductance quantization behavior. Figure 3 shows the typical ranges of the three characteristic length in metal and semiconductor materials. In the devices with atomic-scale CF, the CF configuration is determined by the atomic granularity of the material. In this case, the transport through the CF is governed by the quantum nature of conductance, i.e., the current is carried along the discrete conductance channels. The reason for the occurrence of conductance quantization is that the electrons are not scattered when transporting through the atomic-scale conductor. The conductor behaves like a waveguide for electrons and does not follow the Ohm’s law anymore. The waveguide could be understood as a ballistic transportation path made up of a bundle of discrete conductance channels, with each contributing a maximum amount of one *G*_0_ to the total conductance of the conductor. The total conductance of the conductor is described by the Landauer formula, *G* = *G*_0_∑_*i*_*T*_*i*_ [144]. *T*_*i*_ is the transmission probability of each discrete conductance channel. If the channel is assumed to be fully transmitted, *T*_*i*_ equals to one. *T* = ∑_*i*_*T*_*i*_ is the transmission probability of the whole conductor, which is determined by the details of the conductor geometry and the electronic structure of the conductor material [145].

In fact, the phenomenon of quantum conductance was first observed by Van Wees et al. in a two-dimensional electron gas of a GaAs-AlGaAs heterostructure in 1988 [146]. Similar results were reported almost simultaneously by Wharam et al. [147], also using a 2D electron gas on a GaAs-AlGaAs heterojunction, at 0.1 K. Since then, the phenomenon of quantized conductance has been reported in various physical structures such as atomic quantum contact [68, 146, 148–155], mechanically controllable break junctions [156–160], nanotubes [161–167], and the current-induced local oxidation of nanoscale constrictions [168].

**Fig. 4:**
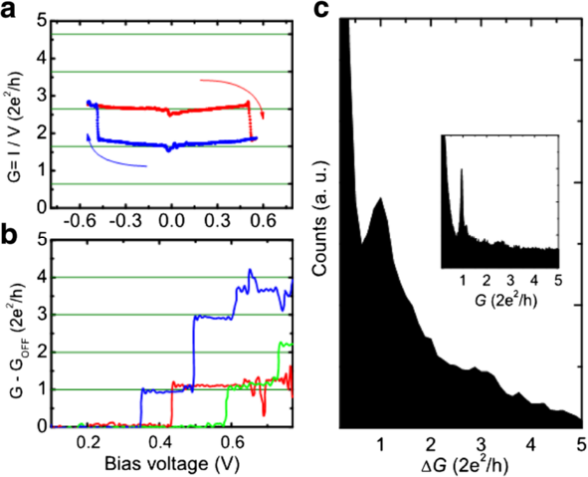
Switching characteristics and conductance quantization observed in nanoscale junctions with a structure of tungsten tip/ionic conductor layer/silver film [191]. **a** Conductance change during SET and RESET operation which shows atomic-scale conductance switching. *Green lines* act as guides to the eye representing a series of conductance levels with equal interval of 1 *G*_0_. **b** Conductance change from high resistance state to low resistance state following voltage sweep for three independent conductance states. **c** Histogram of the conductance difference between high resistance states and the low resistance states during the voltage sweep. The histogram consists of 130 independent *I* − *V* curves with initial ON-state conductance smaller than 10 *G*_0_. Δ*G* is the difference between the conductance and the zero-bias conductance. The inset histogram shows 5000 repeated and closing cycles at a constant bias voltage of 100 mV. Reproduced with permission

**Fig. 5:**
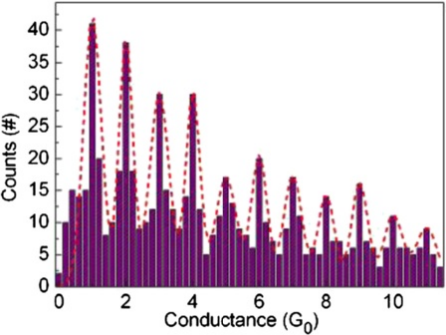
Histogram showing typical conductance quantization phenomenon [171]. The data are extracted from pulse stimuli results of Ti/Ta_2_O_5_/Pt-structured memory cells. The data are grouped in every 0.2 *G*_0_. Histogram consists of 662 conductance values in 22 memory cells. The *dashed curve* which represents Gaussian fitting curve of the histogram acts as a guides to the eye. Reproduced with permission

**Fig. 6:**
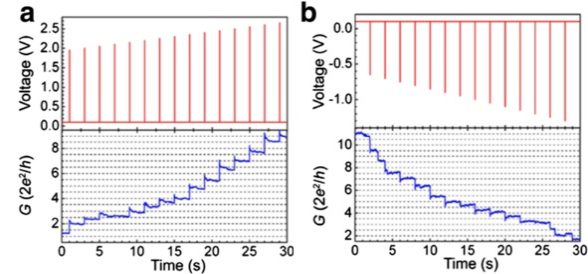
Quantized conductance phenomenon observed by pulse stimuli operation method in Ag/poly(3-hexylthiophene): [6, 6]-phenyl-C61-butyric acid methyl ester/indium–tin oxide sandwich structured devices [183]. Conductance quantization is observed under **a** successive positive pulses and **b** successive negative pulses. Positive voltage pulses are 1 μs wide and negative voltage pulses are 5 ms wide. Two adjacent positive or negative pulses are with an interval of 2 s and an increment of 0.05 V. The conductance is read under a basal voltage of 0.1 V. Reproduced with permission

**Fig. 7:**
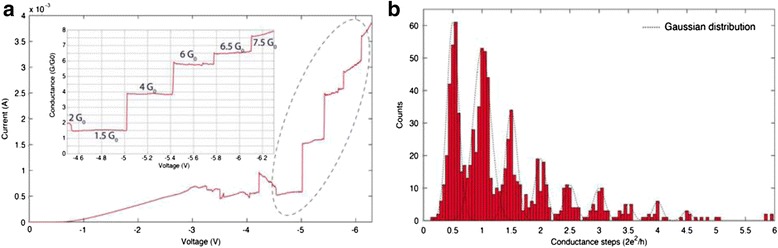
Quantized conductance steps observed in poly-Si/SiO_x_/p-type Si-structured RRAM devices [186]. **a** Current–voltage curves showing nonlinear behavior with inset showing the relation between conductance and voltage. Several conductance quantization levels can be seen at both integer and half-integer multiples of *G*_0_. **b** Histogram consisting of about 1000 conductance steps in which half-integer multiples of *G*_0_ are clearly revealed. A series of Gaussian distributions act as guides to the eye (*dotted lines*). Reproduced with permission

**Fig. 8:**
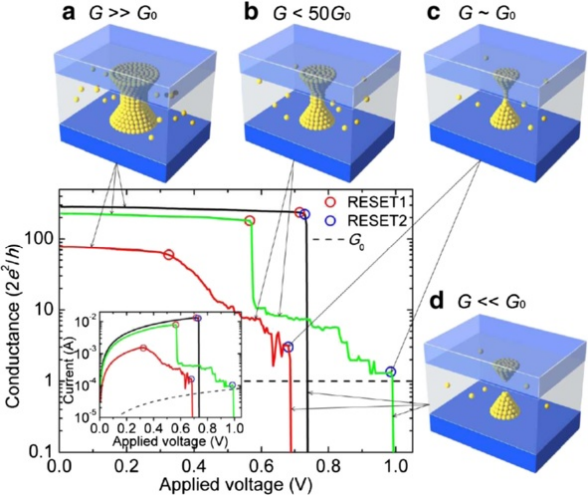
Typical conductance-voltage and current–voltage curves corresponding to RESET process of Pt/HfO_2_/Pt devices [70]. *Black curves* show the usually observed abrupt RESET switching. *Green curves* display several successive jumps and *red curves* show progressive RESET process. Insets *A*–*D* show the different stages of the CF during the RESET process. The quantized conductance states in the step-like or progressive RESET processes are the intermediate states between low and high resistance states. A CF with conductance of the order of *G*_0_ = 2*e* ^2^/*h* is the natural boundary between the LRS and HRS states. The step-like or progressive RESET transition finalizes with an abrupt conductance drop of several orders of magnitude. This final drop corresponds to the opening of a spatial gap (potential barrier) in the CF. Discrete changes of conductance of the order of *G*_0_ recorded during the step-like or progressive RESET transitions are interpreted as the signature of atomic-sized variations of the conducting filament (CF) nanostructure. Reproduced with permission

As conductance quantization effect in RRAM has the potential applications of multi-level storage, it has attracted much attention in recent years. Conductance quantization phenomena were initially found in ECM devices [169] and then in VCM and TCM devices [69, 170, 171]. Conductance quantization effect in RRAM has been studied and reported in a series of literatures [69, 70, 126, 127, 129, 137, 140, 170–190], involving various materials and different RS mechanisms, CF types, SET, or RESET processes, as listed in Table 2. Some typical experimental observation results are given below as illustrations for quantum conductance phenomenon, as shown in Figs. 4, 5, 6, 7, and 8 [70, 171, 183, 186, 191].

**Table 2:** Different material systems showing conductance quantization effect

System	RS mechanism	CF	Quantization level	RS polarity	QC observed in SET or RESET
Ag_2_S or Cu_2_S (vacuum gap) [169]	ECM	Ag	*G*_0_	Bipolar	SET
Ag/AgI/Pt [180]	ECM	Ag	*G*_0_	Bipolar	SET
Ag/SiO_2_/Pt [195, 213]	ECM	Ag	0.5 *G*_0_	Bipolar	SET
Ag/Ta_2_O_5_/Pt [181]	ECM	Ag	*G*_0_	Bipolar	SET and RESET
Ag/Ag_2_S/Pt (STM tip) [182]	ECM	Ag	*G*_0_	Bipolar	RESET
Ag/P_3_HT:PCBM/ITO [183]	ECM	Ag	*G*_0_/0.5 *G*_0_	Bipolar	SET and RESET
Ag/a-La_1-x_Sr_x_MnO_3_/Pt [185]	ECM	Ag	*G*_0_	Bipolar	SET
Ag/ionic conductor-layer/W tip [191]	ECM	Ag	*G*_0_	Bipolar	SET
Ag/GeS_2_/W [193]	ECM	Ag	*G*_0_	Bipolar	SET
Cr/p^+^-amorphous silicon/V [192]	ECM	Metal	0.5 *G*_0_	Unipolar	SET
Nb/ZnO_x_/Pt [170]	ECM	Nb or Vo	*G*_0_/0.5 *G*_0_	Bipolar	SET and RESET
Cu/HfO_X_/Pt [190]	ECM	Cu	0.5 *G*_0_	Bipolar	SET
Pt/HfO_2_/Pt [69]	VCM	Vo	0.5 *G*_0_	Unipolar	RESET
ITO/ZnO_x_/ITO [170]	VCM	Vo	0.5 *G*_0_	Unipolar	SET and RESET
Ti (Ta, W)/Ta_2_O_5_/Pt [171]	VCM	Vo	*G*_0_	Bipolar	SET and RESET
V/V_2_O_5_/V [174]	-	-	0.5 *G*_0_	-	SET
W/CeO_x_/SiO_2_/NiSi_2_ [179]	VCM	Vo	0.5 *G*_0_	Bipolar	RESET
n-Si/SiO_x_/p-Si [186]	VCM	Vo	*G*_0_/0.5 *G*_0_	Bipolar and Unipolar	SET
Ti/HfO_2_/TiN [188]	VCM	Vo	*G*_0_	Bipolar	RESET
Ti/TiO_2_/SrTiO_3_/n-Si [194]	VCM	Vo	*G*_0_	Bipolar	SET

The quantized conductance state of CF is practically an intermediate state or a specific LRS state with the conductance (*G*) in the order of *G*_0_, i.e., integer multiples of *G*_0_. One *G*_0_ can be simply understood as being corresponding to a single atomic point contact or a nanowire. Conductance quantization effect indicates that the evolution of CF can be modulated to be in units of single atomic point contacts.

### Structures, Materials, and Operation Methods of RRAM with QC Effect

There are many observations and reports of quantum conductance phenomenon in RRAM. Different device structures, switching and electrode materials, and operating methods are applied. They are summarized respectively as follows.

#### Device Structures

**Fig. 9:**
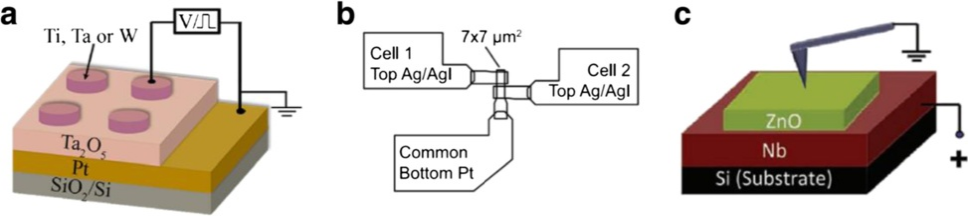
Typical RRAM device structures showing conductance quantization effect. **a** A commonly used sandwich RRAM structure [171]. **b** A crossbar structure [180]. **c** An ultra-small-sized RRAM device using CAFM tip as top electrode [170]. Reproduced with permission

The basic structure of RRAM is a thin resistive switching layer, which is usually nanometers in thickness, sandwiched between two electrodes. Many derivative structures have been fabricated based on this basic structure. There are three kinds of structures of RRAM device exhibiting conductance quantization effect, as shown in Fig. 9. Figure 9a [171] shows a commonly used sandwich RRAM structure. It is a stack of thin films of bottom electrode, RS layer, and patterned top electrode. Figure 9b [180] shows a crossbar structure. Figure 9c [170] shows a structure similar to those in Fig. 9a, b, with the only difference being that a tip, such as a conductive atomic force microscopy (CAFM) tip, is used as the top electrode. More detailed fabrication information is not given here for it is not the main point of this paper.

#### Material Systems

Since the quantized conductance phenomenon is the property of nanoscale conductors, the materials of RRAM devices especially those consisting of the CF play an important role to the quantized conductance behaviors. As has been discussed in “Introduction” section, the types of RS and electrode materials determine resistive switching mechanisms for the filament-based RRAM. For ECM devices, resistive layer is sandwiched between an inert electrode and an active electrode. During the forming and SET process, the metal ions of the active electrode are driven into the resistive layer, forming the CF. For VCM devices, both top and bottom electrode are inert and the CF is consisted of oxygen vacancies. So as for the ECM mechanism, the material of the active electrode is critical for the observation of the quantized conductance, while for the VCM mechanism, the material of the resistive layer plays a more important role.

In fact, the papers on conductance quantization in RRAM were initially published in 1991. Hajto et al. reported their observation of conductance quantization of RRAM for the first time. The studied device structure is Cr/α-Si:H/V [192]. Yun et al. showed similar result in the V/α-V_2_O_5_/V [174] device. Quantized conductance effect was observed in cation migration-based RRAMs [172, 173] and then extended to anion migration-based RRAMs [69, 70, 133, 169, 170]. Since 2012, conductance quantization has attracted a lot of attention, mainly for its potential applications in the multi-level storage, and also for its interesting physics behind the phenomena. As a result, conductance quantization has been reported in more and more RRAM devices, as shown in Table 2. It should be noted that the reports of conductance quantization in sandwich structures with a vacuum gap [169] or a scanning tunneling microscope (STM) tip [170] are also included in this table.

In most cases of ECM RRAMs with QC effect, the reported material for active electrode is Ag [169, 180–183, 185, 191, 193]. Besides Ag, Nb [170] and V [174] have also been reported to be able to form quantum conductors in RRAM. The RS materials involving QC effect include traditional solid-state electrolyte materials such as AgI [180], Ag_2_S [169, 182], Cu_2_S [169], GeS_2_ [193], and transition metal oxides, such as Ta_2_O_5_ [171, 181], LaSrMnO_3_ [185], α-Si [192], and polymer [183]. In bipolar and unipolar VCM devices with QC effect, the reported resistive switching material includes silicon oxide [186], transition metal oxides, such as HfO_2_ [69, 188], Ta_2_O_5_ [171, 181], ZnO_x_ [170], etc., and bi-layered oxides, such as TiO_2_/SrTiO_3_ [194] and GeO_x_/SiO_2_ [179]. Among the reported RS materials which exhibit conductance quantization, Ta_2_O_5_ [171, 181] and HfO_2_ [69, 188] show good performances irrespective of the type of the CFs, i.e., Vo-CF or metal CF. Both ECM and VCM devices made up of HfO_2_ and Ta_2_O_5_ materials have shown obvious QC effect, as shown in Figs. 2, 5 [171], and 8 [70].

It can be seen from Table 2 that most reported devices show conductance step with integer multiples of *G*_0_, while in some material systems, the conductance variation step may be half-integer multiples of *G*_0_. The explanation of this difference will be discussed later in “Theory and Modeling of Quantum Transport in RRAM” section.

#### Operating Methods

To successfully observe the QC effect in RRAMs, it is of importance to make use of appropriate operating methods to the devices to accurately control the size of CFs to be close to the atomic scale. In this section, we will deal with all kinds of reported operating methods, including fresh device operation, voltage sweeping, voltage pulse operation, current sweeping, and constant voltage bias operation.

##### Fresh Device Operation

**Fig. 10:**
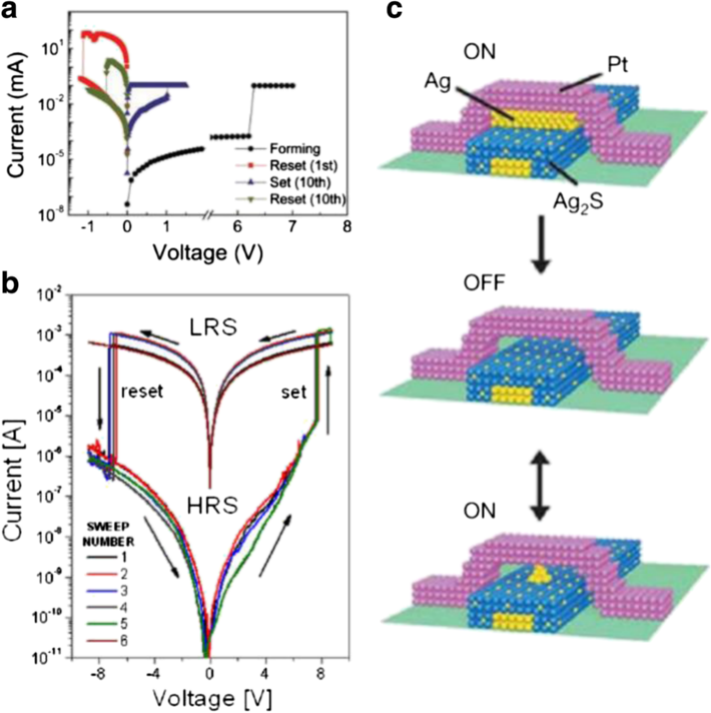
Typical forming, SET, and RESET characteristics of fresh RRAM devices with different initial resistance states. **a** Switching process illustration of Nb/ZnO/Pt device with an initial high resistance state [170]. Higher voltage in forming is needed compared to that in the SET process. **b** Switching process illustration of a free-forming p^+^Si/NiSi_2_/SiO_2_/CeO_x_/W device [179]. The Forming process and the SET process show no obvious difference. **c** Schematic illustration of Ag_2_S-based QCAS device and switching behavior between OFF- and ON-state [169]. The initial state of the device is ON-state and a RESET process is needed to start the switching cycles. Reproduced with permission

Before analyzing the detailed operation methods to achieve QC effect, we need to first discuss the different operating conditions for the RRAM devices with different initial resistance states (IRS). For the fresh RRAM devices, most of them show an initially very high resistance state and a forming operation is needed to form CF in the resistive layer. Since the fresh resistive layer is usually in high resistance state, a much higher voltage, compared with the SET process, is needed to form the CF in the resistive layer, as shown in Fig. 10a [170]. Compared to the forming process, the voltage amplitude applied in the SET process is lower, because the CF formed in the forming process will not be dissolved completely in the successive RESET operation, thus a lower voltage can program the device. But for some RRAMs, the forming process and the SET process show no obvious difference, i.e., the characteristics of the device in initial fresh state and high resistance state have no clear distinction, as shown in Fig. 10b [179]. In other words, this kind of RRAM device has the free-forming characteristics. Some RRAM devices also possibly show an initial low resistance state. For example, in the quantized conductance atomic switch (QCAS), as shown in Fig. 10c [169], it is initially in the ON-state. To start the switching cycles, the device needs to be RESET at first under a certain positive voltage bias. Due to the large amount of Ag atoms to be ionized to incorporate into the Ag_2_S crystal in this first RESET process, the switching time of this process is quite long, lasting for a few seconds. But after this initial RESET operation, the device can work quickly with a high operation frequency of 1 MHz.

##### Voltage Sweeping Mode

**Fig. 11:**
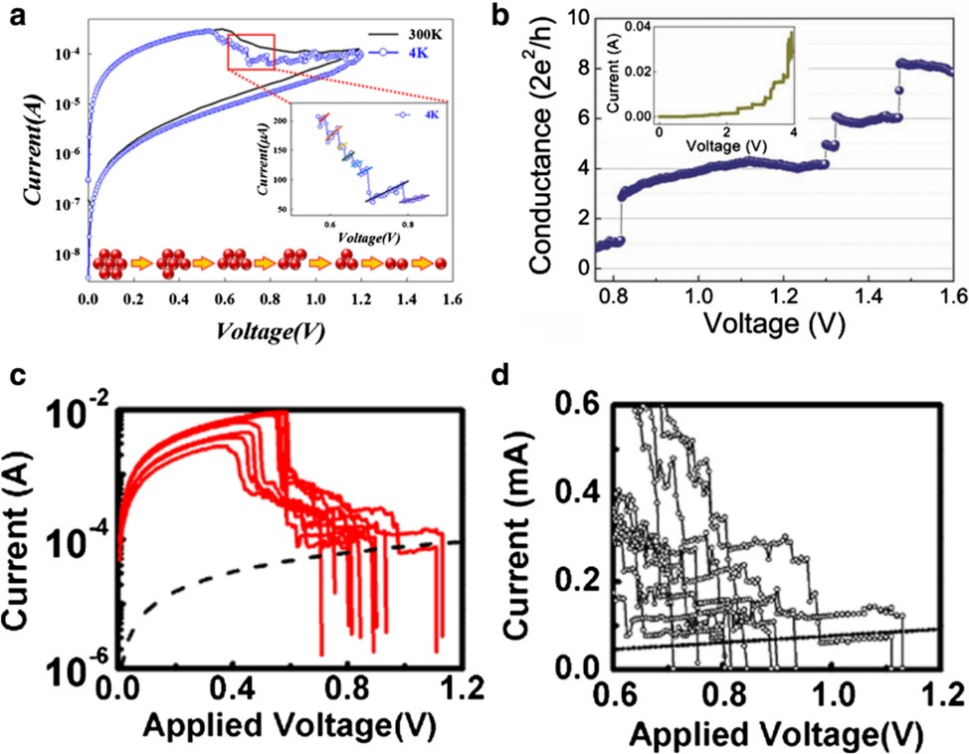
Typical conductance quantization phenomenon observed in different structured devices under voltage sweeping mode. **a** Current jump observed in Ti/HfO_2_/TiN-structured memristor during RESET process. The inset diagram indicates the discrete resistance change due to quantum atomic reaction during RESET process [188]. **b** Conductance quantization observed in Nb/ZnO/Pt during SET process. [170] The inset shows the current–voltage curve in a larger voltage range from 0 to 4 V. **c** Progressive RESET process in Pt/HfO_2_/Pt devices [69]. The *dashed line* corresponds to the current–voltage curve of 1 *G*_0_. **d** Detail of the current–voltage evolution of (**c**). Reproduced with permission

After the initial process which involves forming/SET, RESET or no particular operation method to start switching cycles, a certain operation method is needed to switch the device between ON-state and OFF-state. The most common operation method is voltage sweeping mode. The voltage sweeping in SET process induces electrochemical reactions resulting to the formation of CF. The voltage sweeping in RESET process contributes to joule-heating-assisted oxidation followed by the diffusion of metal ions or oxygen vacancies (Vo) under the concentration gradient and the applied electric field [181]. Many experimental results [69, 170, 171, 179, 180, 188] showing conductance quantization under voltage sweeping mode have been reported. Figure 11 shows some of the reported experimental results in different device structures. Figure 11a [188] shows the progressive RESET process of the Ti/HfO_2_/TiN-structured devices which exhibit bipolar RS behaviors. In the inset of Fig. 11a, zoomed current and voltage relation is shown and several current jumps which indicates the conductance quantization could be clearly seen. Figure 11b [170] shows the conductance change in bipolar characterized Nb/ZnO/Pt devices as a function of the bias voltage during the SET process. At least four conductance jumps are shown in the Fig. 11b and the conductance changed at a step of the integer multiples of quantum conductance *G*_0_ from 1 to 8 *G*_0_. The inset of Fig. 11b shows the current–voltage curve in a larger voltage range from 0 to 4 V. Figure 11c, d [69] shows the current–voltage curves in the RESET process of the unipolar Pt/HfO_2_/Pt devices. Figure 11d shows the detail of Fig. 11c during the last phase of the RESET transients. The dash line in Fig. 11c corresponds to the current–voltage curve of a conductance of 1 *G*_0_.

##### Voltage Pulse Operation Mode

**Fig. 12:**
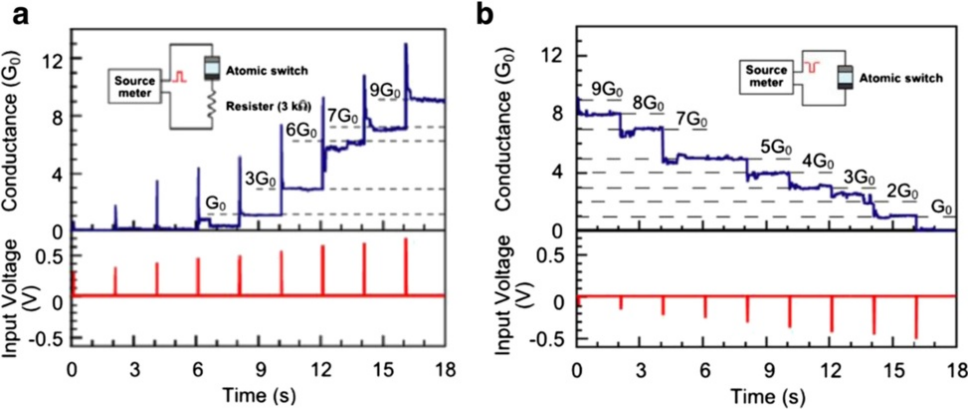
Quantized conductance observed in Ag/Ta_2_O_5_/Pt-structured ECM devices under voltage pulse operation mode [181]. **a** The value of conductance increases at steps of integer multiples of conductance quantum *G*_0_ in the SET process under positive pulses with a width of 20 ms at an interval of 2 s. In order to prevent hard breakdown of RRAM device, a current-limiting resistor of 3 kΩ was connected in series with the device. **b** Quantized conductance decrease phenomenon observed in the RESET process under reversed voltage polarity. No current-limiting resistor is needed in the negative pulse stimuli mode. Reproduced with permission

By applying appropriate pulse voltage, the conductance state of the RRAM device could be changed at steps of quantum conductance. Both ECM and VCM RRAMs show quantized conductance step change under voltage pulse operation, as shown in Figs. 12 [181] and 13 [171], respectively. For voltage pulse operation method, three parameters could be tuned, including the pulse amplitude, pulse width, and time interval between two adjacent voltage pulses.

**Fig. 13:**
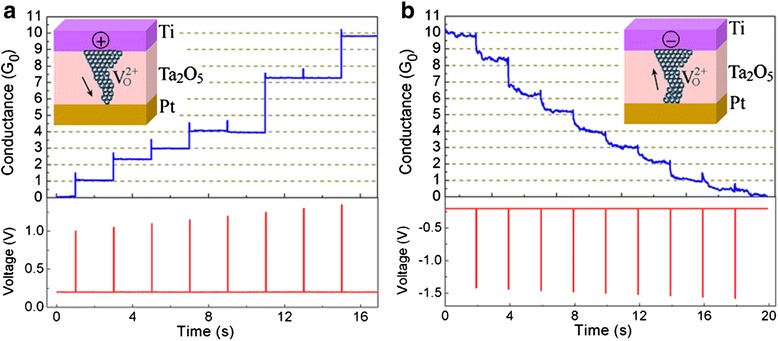
Quantized conductance observed in Ti/Ta_2_O_5_/Pt-structured VCM devices under voltage pulse operation mode [171]. **a** The value of conductance increases at steps of integer multiples of *G*_0_ in the SET process under positive pulses with a width of 100 ns at interval of 2 s. **b** The value of conductance decreases at steps of integer multiples of *G*_0_ in the RESET process under negative pulses with a width of 1 μs at an interval of 2 s. Reproduced with permission

Take Ag/Ta_2_O_5_/Pt RRAM as an example. Figure 12 shows the QC effect observed in this device. Under the pulse operation mode, the switching speed of the device is of the magnitude of μs to ns which is related to the values of the high resistance and the input pulse. The switching time is much shorter than the reaction time of the current compliance function, which results in the ineffectiveness of the current compliance function and leads to the further growth of the CF even after the current reaches the value of the compliance current. The solution for this problem is to insert a 3 kΩ resistor in series with the RRAM device, as shown in the inset of Fig. 12a. The current is limited by a 3 kΩ resistor during the stage that the RRAM switches from high resistance state to low resistance state. It is very important to limit the current value when the SET transition occurs. If there is no current limitation, the conductance of the device will abruptly jump to about 20 *G*_0_, which indicates that the formed filament is rather thick and robust and quantized conductance step disappears. As can be seen from Fig. 12a, the quantized conductance increases from 0 to 9 *G*_0_ at a step of conductance quantum under a series of increasing pulses from 0.3 to 0.7 V at a step of 0.05 V with time intervals of 2 s. Negative pulses with the same pulse width and interval time from −0.1 to −0.5 V were applied after the successive positive voltage pulses. In the negative pulse operation which corresponds to the RESET process, no series resistor is needed since the current in the circuit will decrease as the resistance value of RRAM increases in the RESET process. Similar quantized conductance change behavior has also been reported in the VCM RRAM with a structure of Ti/Ta_2_O_5_/Pt, as shown in Fig. 13.

**Fig. 14:**
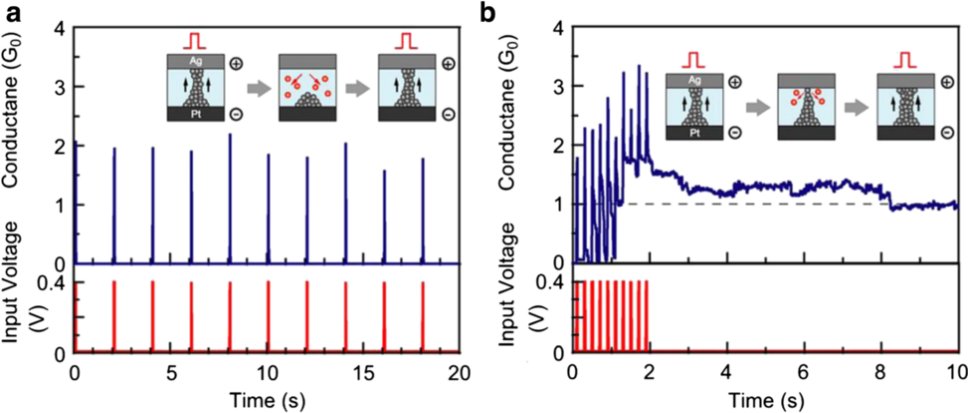
Quantized conductance change behavior under different time intervals [181]. **a** The conductance change under ten successive voltage pulses of 0.4 V with a pulse width of 20 ms at intervals of 2 s. The conductance state could increase to about 2 *G*_0_ under the input pulses but immediately decays to zero after each input pulse is completed. **b** The conductance evolution under ten successive voltage pulses of 0.4 V with a width of 20 ms at intervals of 0.2 s. In this case, the conductance gradually increases and maintains at about 1 *G*_0_ for more than 60 s after the tenth input pulse. Reproduced with permission

The quantized conductance states change not only depending on the amplitude of the pulses but also depending on the time interval of the adjacent pulses. As shown in Fig. 14 [181], successive pulses with sufficiently long interval do not obviously change the conductance state. Whereas at short interval, pulses of the same amplitude and width make the conductance temporarily increase and gradually reach a constant value of *G*_0_.

##### Current Sweeping Mode

**Fig. 15:**
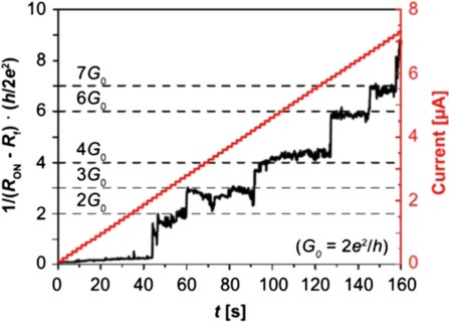
Quantized conductance phenomenon observed in Ag/AgI/Pt devices under current sweep mode [180, 195]. It can been seen that more than five resistance levels which are integer multiples of conductance quantum *G*_0_ have been observed. Reproduced with permission

In some material systems, no more than one or two discrete conductance drops could be detected by conventional voltage sweep operation. Current sweep mode is utilized as an alternative operation mode. Through this method, more discrete conductance levels could be observed, as shown in Fig. 15 [180, 195]. The different quantization evolution behavior in SET process by taking voltage sweep mode and current sweep mode comes from the different formation process of the CF. In the voltage sweep mode, stepwise increased voltage is applied to the device. As the voltage increases to the SET point, the filament forms and the resistance of the device suddenly drops to a much lower value. This leads to an abrupt current jump and the switching time is faster than the response time of the current compliance current which causes a further growth of CF after the SET point. Both voltage and current increase at the SET point which results in a positive feedback to the CF formation, thus leading to a very fast CF formation process. Whereas in the current sweep mode, the current is programmed to increase stepwise. At the SET point, the voltage dropped across the device decreases abruptly, due to the sudden decrease of the device’s resistance. This leads to a negative feedback to the CF formation, so the CF formation process is more gradual than that under voltage sweep. In this case, more discrete conductance levels could be observed.

##### Constant Voltage Bias Mode

**Fig. 16:**
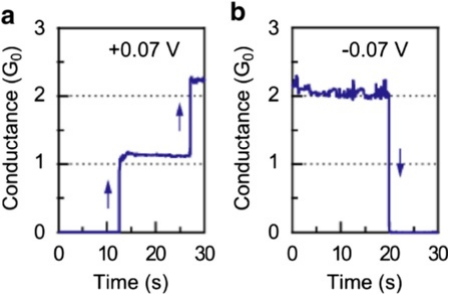
Conductance jump at the integer multiples of *G*_0_ under voltage bias in Ag/Ta_2_O_5_/Pt device [181]. **a** Conductance increase under positive voltage bias of +0.07 V. **b** Conductance decrease under negative voltage bias of −0.07 V. Reproduced with permission

**Fig. 17:**
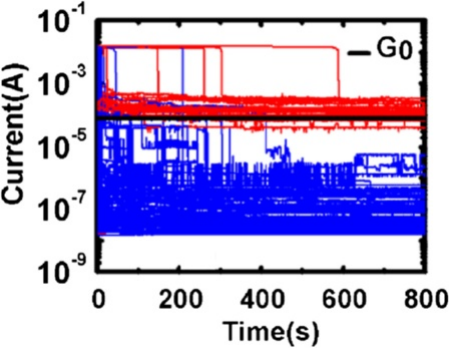
Current evolution of Pt/HfO_2_/Pt device under constant voltage bias for RESET process indicating quantum level change of the conductance [69]. Reproduced with permission

Constant voltage bias is another way to observe quantized conductance. The bias value of voltage is critical to the observation of conductance quantization phenomenon. If the voltage amplitude is too large, the device may easily break down or the switching time may be too short to detect. On the other hand, if the amplitude of bias voltage is too small, the switching time of the device may be too long which is time consuming to observe conductance quantization or even no switching behavior could be observed, since the voltage is too small to drive the switching action to occur. Therefore, an appropriate bias voltage amplitude is needed for the observation of conductance quantization phenomenon, as shown in Figs. 16 [181] and Fig. 17 [69].

### Theory and Modeling of Quantum Transport in RRAM

Systems whose dimensions are much larger than microscopic objects like atoms but smaller than macroscopic objects are called mesoscopic systems [196]. When the dimension of the conductor is comparable to or smaller than the electron mean free path (mesoscopic scale), the classical Ohmic transport mechanism breaks down. When the conductor is under sufficiently high voltage, the Ohm’s law does not apply for it either. When the above two conditions are satisfied, ballistic transportation begins to be dominant and the conductor behaves as an electron waveguide [197]. When the transmission probability *T* for the waveguide equals one, this waveguide or conduction channel contributes an amount of *G*_0_ to the total conductance of the mesoscopic conductor.

**Fig. 18:**
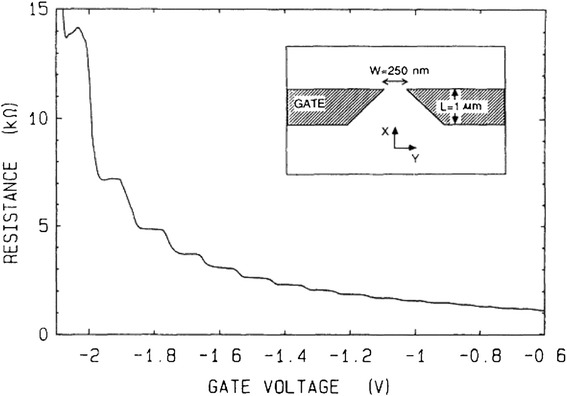
First observation of conductance quantization by Van Wees et al. [146]. The resistance of the point contact is a function of gate voltage at 0.6 K. The electron gas under the gate is depleted at −0.6 V when electrons only transport through the point contact and the contact is fully pinched off at −2.2 V. The inset shows the layout of the point contact. Reproduced with permission

The theory of electron transport mechanism in mesoscopic systems exhibiting quantized conductance has been gradually established through a time span of decades from the suggestion of the prototype by Rolf Landauer in 1957 [198] to the publication of the book “Electronic Transport in Mesoscopic Systems” by Datta in 1995 [143]. Many theoretical calculations about quantum conductance using different models have been reported [197, 199–209]. The quantized conductance steps were obtained in numerical and analytical calculations in a wide variety of materials [144, 205, 206, 208–211]. Review articles summarizing quantized conductance phenomenon in atomic-sized conductors [148] and nanowires [149] have also been published. At the same time, many experiments were also carried out to study quantized conductance [148, 156, 162, 163]. The conductance quantization effect was first observed in ballistic point contacts in the 2DEG of high-mobility GaAs-AlGaAs heterostructures in 1988 by Van Wees et al. [146], as shown in Fig. 18.

In RRAM, many theoretical works concerning calculation and simulation were reported. Several models have been proposed to describe and calculate the ballistic transportation and conductance quantization phenomenon in RRAM devices. As mentioned above, two conditions, i.e., small conductor dimension and high voltage bias, lead to ballistic transportation and conductance quantization, so in all proposed models, either the CF in resistive layer has been considered an atomic-sized conductor in insulator or the most constrictive part in CF is treated as being of atomic size. In this section, we will summarize four models. In the first one, the CF formed in the resistive layer is regarded as a one-dimensional linear atomic chain [175]. The second model treated the narrowest part of a CF as an atomic contact and calculated the electrical transport based on Landauer theory [179, 186, 212]. The third model focuses on CFs consisted of oxygen vacancies and calculated the quantized conductance of the filament with different space of oxygen vacancies through first principle calculation [69]. The fourth model is a circuit model, which ascribes the quantized conductance of the atom point contact in the ECM device to the discharge of the thermal emf voltage [195, 213].

#### One-Dimensional Linear Atomic Chain Model

In the work of Jameson et al. [175], a model based on one-dimensional metal atomic chains was proposed to relate to the quantized conductance phenomenon and to calculate the programming time of RRAM device whose resistance tends to be programmed to integer multiples of fundamental conductance *G*_0_ under sufficiently high currents. This model is different from other previous models which considered the filament as a bulk-like (although small) object whose resistance is continuous and proportional to the bulk resistivity.

**Fig. 19:**
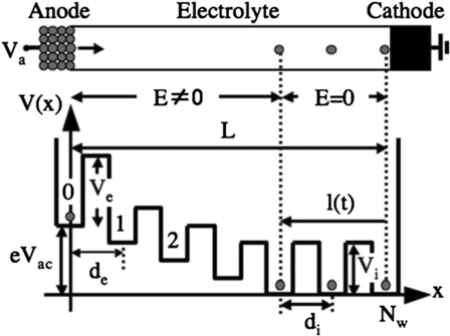
One-dimensional potential model for the forming process of a fresh RRAM cell [175]. The calculation was carried out under the assumption that the electric field *E* is zero within the filament and constant between the anode surface and the tip of the filament. The filament was treated as a one-dimensional atomic chain. Reproduced with permission

In this model, the filament is simply treated as a linear chain of atoms, with the conductance in the order of *G*_0_. A schematic illustration of the model is shown in Fig. 19. The CF is assumed to form from cathode to anode since the metal cations are usually reduced at the cathode. The resistive layer where the CF is formed is treated as a one-dimensional series of *N*_*w*_ potential wells with a uniform height *V*_*i*_ and width *d*_*i*_ (“*i*” for internal). The last atom of the metal atom chain corresponds to the surface of the cathode. The resistive layer where CF is not formed yet is treated as a bulk electrolyte, and the resistive layer is separated by an “emission” barrier of height *V*_*e*_ and width *d*_*e*_ (“*e*” for emission) from the anode surface. When the positive forming or SET voltage *V*_ac_ is applied to the anode, the emission well is raised to an energy of *eV*_ac_, while the well *N*_w_ is kept at zero. A metal ion with a charge of +*e* (e.g., Ag^+^ for Ag/GeS_2_/W cells) will be emitted from anode into the potential well by thermal excitation over the emission barrier. Then, it travels through the periodic potential barriers which represent the resistive layer with no CF inside and stacks up against the cathode to promote the stretch of the CF, i.e., an atomic chain that grows with time. When all *N*_w_ wells are filled with metal ions, the resistance state of RRAM will suddenly switch to low resistance state and the time needed is the programming time of the device.

The programming time (*t*_*p*_) of a RRAM cell whose resistance tends to increase to the integer multiple of *G*_0_ was calculated. It was found that several intrinsic material parameters, including *V*_e_, *d*_*i*_, *W*_*a*_ − *W*_*c*_ (the difference between the work functions of the anode and cathode), influence the programming time of the virgin Ag/GeS_2_/W cells. After selecting proper values for these parameters, the model can nicely describe the dependence of *t*_*p*_ on voltage, temperature, and GeS_2_ thickness (for thick layers), which is in close relation to the quantization of the ON-state conductance. The model is effective for the cases of both constant voltage and ramped voltage programming. Further experimental study of the conductance quantization was reported by the same group in Ag/GeS_2_/W RRAM device afterwards [193] and the result is listed in Table 2.

#### Quantum Point Contact Model

**Fig. 20:**
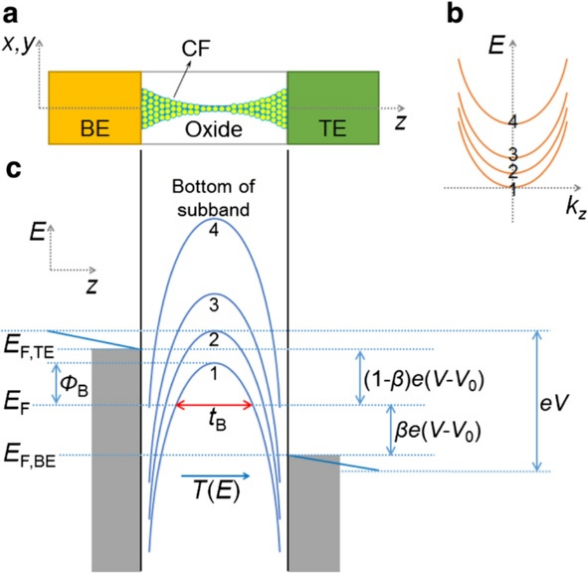
The schematic of the RRAM device with a narrow CF and the corresponding energy band diagram of the quantum point contact model. **a** Schematic structure of the RRAM device with a narrow CF. **b** The dispersion curves of the first four electronic subbands under the confinement of CF in certain *z*. **c** The dependence of the energy level of the bottom of the subbands on *z*. The transmission probability *T*(*E*) of the bottom of ground quantized subband of a parabolic potential barrier is used for the calculation of electrical transport. The *shaded regions* are the states occupied by electrons. The number of the subbands is *N*_ch_, with each one contributing to a conducting mode. In this figure, four subbands are shown. *V* is the applied voltage. *V*_0_ is the voltage dropped on TE and BE, represented by the *two blue oblique lines*. Since *V*_0_ is much lower than *V*, usually it can be neglected in the calculation. *β* is the fraction of voltage that drops at the BE interface, *E*_*F*_ is the Fermi level, *E*_*F*,TE_ and *E*_*F*,BE_ are the TE and BE quasi-Fermi levels, *t*_*B*_ is the width of the potential barrier at the equilibrium Fermi energy (*E* = 0), and *Φ*_*B*_ is the height of the potential barrier, i.e., the bottom of the first subband. The barrier height is different between high resistance state and low resistance state, which leads to different current expressions. In the deep OFF-state, the barrier thickness *t*_*B*_ is equal to the gap length *t*_gap_

To deal with the post-breakdown (BD) conduction of gate dielectric of field effect transistor (FET), Suñé and Miranda have established the quantum point contact (QPC) model [214]. Recently, it has been found that the QPC model can be also made use of to describe the conduction of high and low resistance state in RRAM [71, 179, 182, 212, 215–219]. This model treats the thinnest part of the CF as a quantum point contact. It is able to explain the conductance quantization phenomenon in RRAM. The schematic illustration of the QPC model is shown in Fig. 20 [212]. The thinnest part of the CF is made up of a few atoms (Fig. 20a) and modeled as a potential barrier with several quantized subbands.

In the CF described by the QPC model, it was demonstrated that the Schrodinger equation can be decomposed into the transverse and longitudinal equations. Then, the quantum transport through a 3D tube-like constriction becomes a simple 1D tunneling behavior. The dispersion curve of the electronic subbands could be expressed as:

1 $$ E\left({k}_z,z\right)={\epsilon}_n(z)+\frac{\hslash^2{k}_z^2}{2m} $$

where *z* and *k*_*z*_ are the coordinates in the longitudinal direction in real-space and *k*-space, respectively, *m* is the mass of the electron, and *ℏ* is the reduced Planck constant. If the confinement is in a rectangle shape, then [202]

2 $$ {\epsilon}_n(z)=\frac{\pi^2{\hslash}^2}{2m}\left(\frac{n_x^2}{L_x{(z)}^2}+\frac{n_y^2}{L_y{(z)}^2}\right), $$

where *L*_*x*_(*z*) and *L*_*y*_(*z*) are the dimension of the constriction and *n*_*x*_ and *n*_*y*_ are integers. It means that the dispersion curve (*E* − *k*_*z*_ relationship) consists of discrete parabolic subbands in each *z*, as shown in Fig. 20b. *ϵ*_*n*_(*z*) strongly depends on the thickness of the filament. Thinner filament has stronger spacing out of the subbands.

The potential barrier for conducting transmission in the longitudinal direction (*z*) lies in the bottom of the subbands. When the filament is very thick, which means the confinement is weak, the dispersion curve will turn into the 3D bulk energy band. In that case, the bottoms of the subbands are in rather deep energy levels. Whereas, when the filament is very thin, the bottom of the subbands will be lifted. Consequently, the dependence of the energy level of the bottom of the subbands on *z* in a constricted tube is arch-shaped curves, as shown in Fig. 20c. The number of the subbands is just that of the conducting modes or conducting channels *N*_ch_. *Φ*_B_ is the height of the first subband. The barrier width *t*_*B*_ is defined as the width of the first subband at *E* = 0. If the CF is very thick, the barrier disappears. On the contrary, in the HRS state, the CF is ruptured and there is a gap in the CF region, so the barrier becomes very high. In the conduction, the injected electrons need to travel through the potential barrier, with a transmission probability *T*(*E*).

In QPC model, based on the Landauer theory, the current flowing through the RRAM device can be calculated as [11]

3 $$ I=\frac{2e}{h}{N}_{\mathrm{ch}}{\displaystyle {\int}_{-\infty}^{\infty }T(E)\left[f\left(E-\beta eV\right)-f\left(E+\left(1-\beta \right) eV\right)\right]dE}, $$

where *E* is the energy, *f* is the Fermi-Dirac distribution function, and *N*_ch_ is the total number of 1D opened conducting channels connecting the electrodes. An inverted parabolic potential barrier is assumed to get an analytical expression for the transmission probability [71]:

4 $$ T(E)={\left\{1+ \exp \left[-{\alpha}_B\left(E-{\varPhi}_B\right)\right]\right\}}^{-1}, $$

where *α*_*B*_ is related to the inverse of potential barrier curvature and is proportional to the thickness of the barrier, i.e., ${\alpha}_B={t}_B{\pi}^2{h}^{-1}\sqrt{2{m}^{*}/{\varPhi}_B}$ [71, 214]. m* is the effective electron mass. Inserting Eq. (4) into Eq. (3), we can get

5 $$ I\approx \frac{2e}{h}{N}_{\mathrm{ch}}\left\{ eV+\frac{1}{\alpha}\mathrm{L}\mathrm{n}\left[\frac{1+ \exp \left\{{\alpha}_B\left[{\varPhi}_B-\beta eV\right]\right\}}{1+ \exp \left\{{\alpha}_B\left[{\varPhi}_B+\left(1-\beta eV\right) eV\right]\right\}}\right]\right\} $$

Equation (5) is applicable for both HRS and LRS, with the difference in the values of *α*_*B*_ and *Φ*_*B*_ which represent the difference in potential barrier. In HRS, there is a gap in the CF region, so at low applied voltages (i.e., *V* → 0), Eq. (5) can be simplified as

6 $$ I \cong {N}_{\mathrm{ch}}{G}_0 \exp \left(-{\alpha}_B{\varPhi}_B\right)V. $$

So, the conduction in HRS is just determined by the barrier through the parameters *α*_*B*_ and *Φ*_*B*_ [215, 216, 219]. While in LRS, there is no spatial gap, so Eq. (5) converges to

7 $$ I \cong {N}_{\mathrm{ch}}\beta {G}_0V, $$

which is a linear *I*–*V*, consistent with that usually observed in LRS. When the CF is very narrow, i.e., when *N*_ch_ is small, Eq. (7) accounts well for the experimentally observed conductance quantization effects. The CF conductance is expressed as

8 $$ G \cong {N}_{\mathrm{ch}}\beta {G}_0, $$

showing that *G* is just the integer multiples of the quantum of conductance *G*_0_, when the voltage drop at two interfaces is asymmetric, i.e., *β* = 1. If *N*_ch_ is large, the model approaches the classical Ohmic regime, where quantization effect is less evident since CF conductance is high.

It is worth noting that there are some amounts of experimental points whose conductance is smaller than *G*_0_, according to the reported results as shown in “Conductance Quantization in RRAM” and “Structures, Materials, and Operation Methods of RRAM with QC Effect” section. Values slightly different from *G*_0_ are possible even when a continuous conducting channel connects the electrodes, since in Eq. (8), *N*_ch_ is an integer whereas 0 < *β* < 1. In an atomic-scale conducting CF or quantum wire (QW), the voltage mainly drops at the interfaces with the external reservoirs and the value of *β* is the fraction of voltage that drops at the BE interface. The value of *β* might change with the actual geometry of the CF and with its coupling to the reservoirs. The presence of impurities in the QW or non-adiabatic coupling with the reservoirs might also explain a conductance smaller than *G*_0_ for each conducting mode [153]. The adsorbed impurities on or in atom chains would change the CF constriction configuration and influence the electronic band structure.

**Fig. 21:**
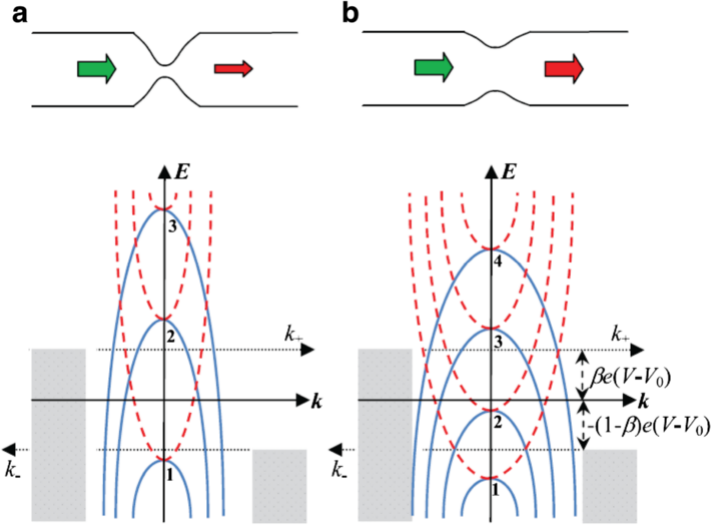
*E–k* relationship for **a** narrow and **b** wide constriction, respectively [179]. The *solid* and *dashed lines* correspond to the bottom of the longitudinal and transversal subbands, respectively. The *shaded regions* are the states occupied by electrons. The *indices* indicate the subband number. A tighter constriction leads to higher energy levels. Reproduced with permission

In a subsequent work, Miranda et al. proposed a simple current–voltage model based on the quantized constriction of RRAM (Fig. 21) and explained the minimum unit of conductance of 0.5 *G*_0_. The left-going current *I*^−^ and right-going current *I*^+^ were respectively calculated as:

9 $$ {I}^{+}=\frac{2e}{h}{\displaystyle {\int}_{-\infty}^{\infty }T(E)M(E)f\left(E-\beta eV\right)dE} $$

and

10 $$ {I}^{-}=\frac{2e}{h}{\displaystyle {\int}_{-\infty}^{+\infty }T(E)M(E)f\left[E+\left(1-\beta \right) eV\right]dE,} $$

where *M* represents the number of conduction modes, and the voltage dropped in electrodes *V*_0_ is neglected. The total current *I* = *I*^+^ − *I*^−^ is given as:

11 $$ I={G}_0\left[\beta {N}^{+}+\left(1-\beta \right){N}^{-}\right]V, $$

where *N*^+^ and *N*^−^ are the number of right-going and left-going conduction modes, respectively, i.e., the number of subbands with *E* ≤ *βe*(*V* − *V*_0_) and *E* ≤ − (1 − *β*)*e*(*V* − *V*_0_) in Fig. 21, respectively. For simplicity, considering the case of a symmetric potential drop at two ends of the constriction, *β* equals 0.5, thus Eq. (11) indicates that when the difference of *N*^+^ + *N*^−^ is an odd number, the conductance values of half-integer multiples of *G*_0_ appear. Simulation results fit well with the experimental results measured in W/CeO_x_/SiO_2_/NiSi_2_ devices [179].

A similar model was proposed by Mehonic et al. [186]. This model treats filaments as quantum constrictions within the framework of Landauer theory. In this model, the potential drop on the two ends of the constriction is not assumed to be symmetric.

**Fig. 22:**
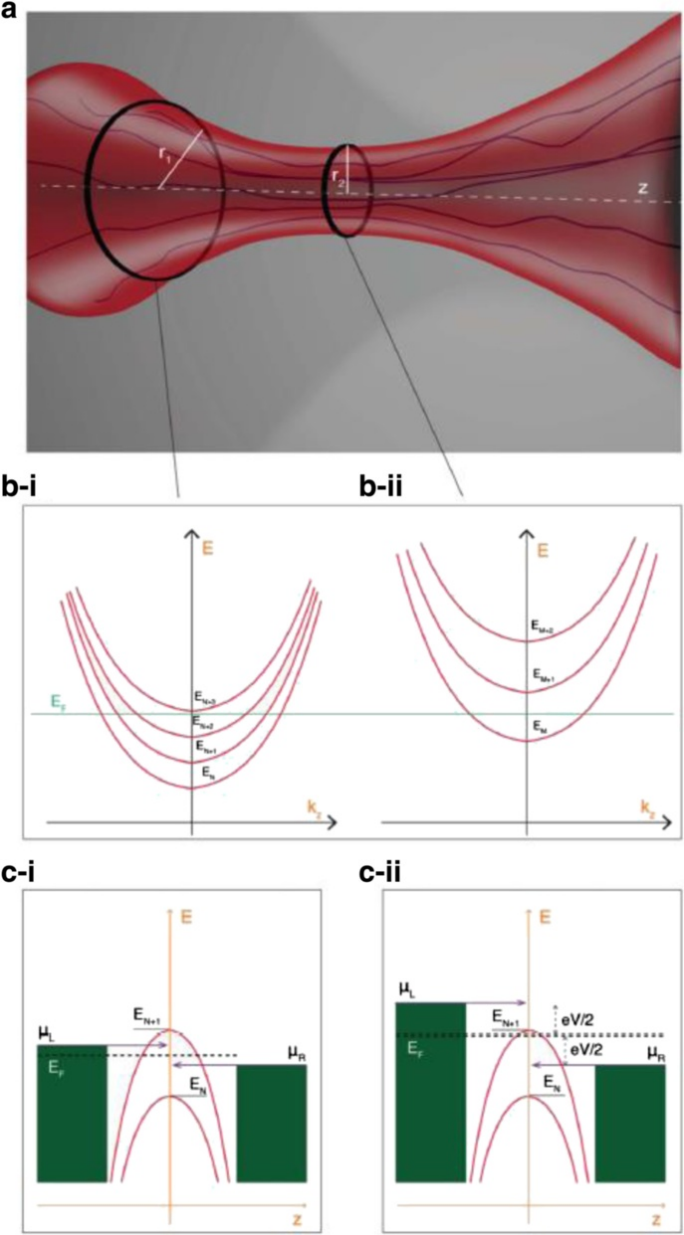
Quantized conductance effect based on the quantum point contact model [186]. **a** Schematic illustration of a conducting filament with a lateral constriction of one or several atoms at the narrowest part of the filament. **b**-**I** Dispersion curves of the first four electronic subbands at the edge of the constriction. **b**-**II** Dispersion curves of the first three subbands at the center of the constriction where the confinement is stronger which leads to a spacing out of the subbands. **c**-**I** When the difference in chemical potential between the left and right reservoirs is small, both the left-going and right-going electron modes fall within the same subband. **c**-**II** When the difference in chemical potential between the two reservoirs is large, the left-going and right-going electron modes fall into different subbands. Reproduced with permission

The schematic illustration of this model is shown in Fig. 22. The lateral confined quantum constriction for carriers to flow through produces a set of discrete one-dimensional subbands in the conduction band. More conduction modes are allowed if the size of the constriction increases. Half-integer quantum conductance was studied under the quantum point conduction model. By assuming the transmission probability to be one for electrons with energy above the minimum energy value of the subband, to be zero with energy below this, and adopting the zero temperature limit, the total current is

12 $$ I={I}_R-{I}_L=\frac{1}{2}\left({N}_R+{N}_L\right){G}_0V, $$

where *N*_*R*_ and *N*_*L*_ are the numbers of occupied subbands accessed from the right and left sides, respectively. Here, symmetric voltage drop in the TE and BE interfaces is assumed, i.e., *β* = 0.5. Then, the half-integer quantum value appears when *N*_*R*_ + *N*_*L*_ is an odd number, which is in coherence with the result reported by Miranda et al. as described above. In all ECM devices, since the electron reservoirs are highly conductive, it is impossible to maintain a large difference in chemical potential, which is the reason for the difficult observation of half-integer quantization. While in another case, i.e., in VCM devices, most of them exhibit half-integer quantization. An important conclusion drawn in this paper is that the key quantity governing the type of quantization is the difference in chemical potentials between the two reservoirs.

The appearance of half-integer multiples of quantized conductance might also arise from the absence of the spin degeneracy. The quantum of conductance *G*_0_, i.e., 2*e*^2^/*h*, is equally contributed by two spin-degenerate transport channels. Thus, in nonmagnetic materials where the spin degeneracy is reserved, the conductance is the integral multiple of *G*_0_. While when the spin degeneracy is broken in magnetic systems, a single spin channel will contribute a conductance of 0.5 *G*_0_, i.e., *e*^2^/*h*, leading to the conductance of half-integral multiple of *G*_0_. As easily inferred from Table 2, the half-integer *G*_0_ appears mostly when the CF is composed of Vo. That is because the Vo can carry a weak magnetism in some cases, according to lots of previous studies [41, 220]. The magnetic CF may bring spin-splitting conductance channels, which give rise to the observed 0.5 *G*_0_.

#### First Principle Calculation on the Quantized Conductance of Oxygen Vacancy Conductive Filament

**Fig. 23:**
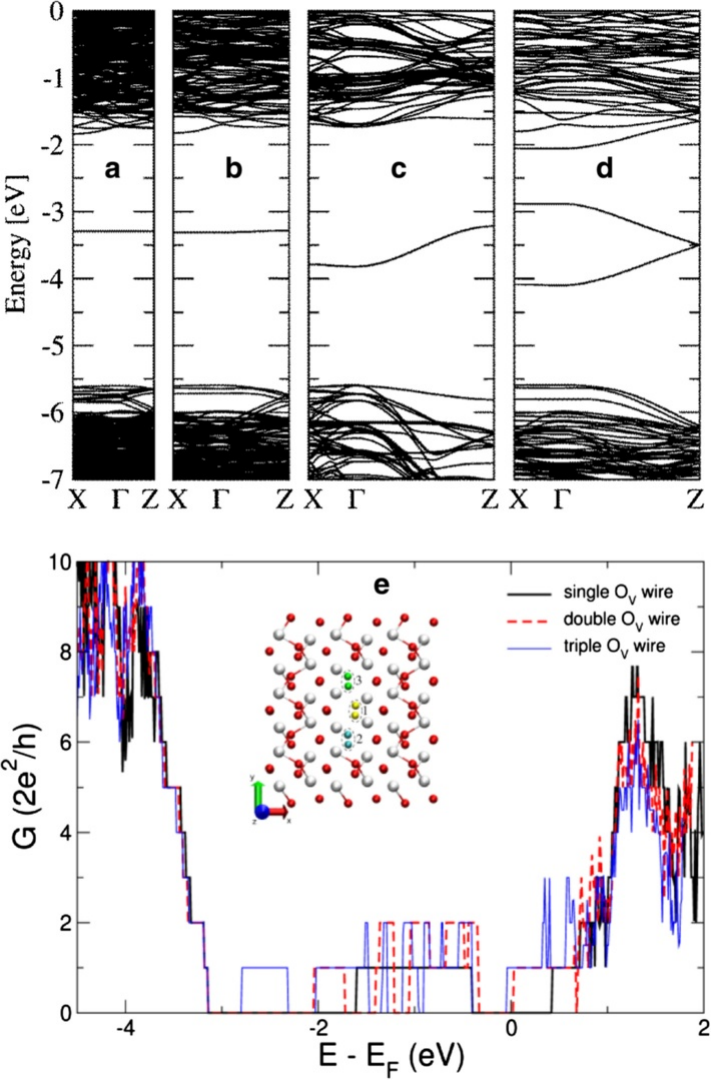
Calculated band structures for crystalline m-HfO_2_ with O vacancies and the corresponding conductance [69]. **a**–**d** Crystalline m-HfO_2_ band structure with different oxygen vacancies separated by 4 *a*_0_, 2 *a*_0_, *a*_0_, and *a*_0_/2. *a*_0_ is the length of the c-axis vector for the m-HfO_2_ primitive cell (0.5296 nm). **e** Conductance as a function of energy corresponding to a HfO_2_ matrix where one, two, or three O atom rows are removed. The rows subsequently removed are shown in the instate (marked as “1,” “2,” and “3”), where *red* and *white spheres* correspond to O and Hf atoms, respectively. Reproduced with permission

As mentioned above, before switching to the high resistance state, the CF in RRAM behaves as a nanoscale conductive path with a few defects such as oxygen vacancies. It is necessary to explore whether the oxygen vacancy paths can explain the quantized conductance behavior. First principle calculations based on the density-functional theory (DFT) were carried out to get the quantized conductance of oxygen vacancy conductive path in crystalline HfO_2_ matrix [69]. In this work, the zero-bias transmission probability *T*(*E*) was calculated by using non-equilibrium Green’s functions. The ballistic conductance was calculated through first principle method based on Landauer theory. The conductance of CF was then calculated via the Landauer formula, *G* = *T*(*E*)*G*_0_. The generation of an oxygen vacancy is considered as the removal of an oxygen atom in a monoclinic-HfO_2_ host. As a result, a filled impurity state is introduced in the band gap which is far from the band edges, as shown in Fig. 23a. The spatial spread of the impurity wavefunction determines that the states overlap between two neighboring oxygen vacancies, which further determines whether the carrier transport is hopping or band transport. As shown by the band structures of monoclinic-HfO_2_ with a chain of oxygen vacancies in Fig. 23a–d, when oxygen vacancies are closer together, the overlap between the impurity wavefunctions increases, thus the impurity band width also increases. Therefore, the transition from hopping to band transport will occur, with a critical oxygen vacancy concentration of about 1.5 × 10^21^ cm^−3^ corresponding to a local composition HfO_2 – x_ with *x* = 0.05. Figure 23e shows the effect of atomic-sized changes in the CF diameter on the obvious change of the CF conductance. When one to three oxygen vacancy columns are formed, as shown by the inset of Fig. 23e, the conductance is observed to increase stepwise, with each transmitting channel contributing a quantum of conductance *G*_0_. This result is in qualitative agreement with the interpretation that the filament with single- to few atom diameters behaves as a quantum wire and the observed conductance quantization originates from single or few atom changes in the atomic structure of the filament.

#### Equivalent Circuit Model for ECM Device Showing Conductance Quantization Effect

**Fig. 24:**
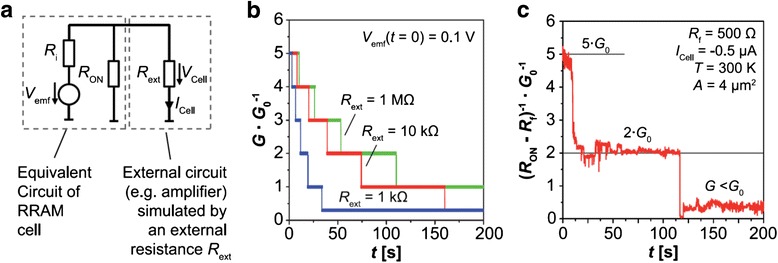
Equivalent circuit model for ECM device [195]. **a** Equivalent circuit model for an ECM cell including a nanobattery *V*_emf_ with an external circuit. *R*_i_ is the total resistance of the ionic current path. *R*_ext_ is the external resistance, e.g., from the neighboring cells in an array or a sense amplifier. **b** SPICE simulation results showing a staircase-like change of the cell conductance resulted from the discharging of *V*_emf_. **c** Evolution of the conductance of a Ag/SiO_2_/Pt cell in crossbar structure under a negative cell current *I*_cell_. Reproduced with permission

Valov and Waser et al. have found that the ECM device is inherently controlled by the non-equilibrium states, which are induced by several factors, including the chemical processes such as the dissolution of the active electrode materials into the electrolyte, the electrochemical processes, and the charge redistribution during operations [221]. The most distinct effect brought out by the non-equilibrium states is the generation of the electromotive force (emf) in the device, suggesting the presence of a nanobattery inside the device. On the basis of this work, recently they proposed a circuit model (Fig. 24a) for the ECM device accounting for quantized conductance [195, 213]. The discharging of the internal emf voltage (*V*_emf_) can influence the device characteristics including ON-resistance (*R*_ON_). In the model, they assumed that in the ON-state with atom point contact, the ON-resistance is restricted in the contact regime, being *R*_ON_ = *R*_c_ = *nG*_0_^− 1^, and assumed that *R*_ON_ is directly dependent on the internal emf voltage ((*V*_emf_)). Figure 24b shows the simulation results about the impact of the external resistance *R*_ext_ on *R*_ON_, exhibiting the staircase-like change of the cell conductance, which well accounts for the experimental results as shown in Fig. 24c. Moreover, the calculated time constants also fit the experimental data as shown in Fig. 24c quite well.

### Prospects of Conductance Quantization in Applications

In these years, the conductance quantization phenomenon in RRAM has been widely investigated and developed, turning into an attractive and well-known effect. From the reported results on the conductance quantization in RRAM, the filament can be of atomic dimensions at the narrowest constriction, which shows that CF-type RRAM devices can still remain functional even if the diameter of CF scales down to the atomic size. Therefore, the ultimate scalability of RRAM is significantly higher than any current mainstream and emerging non-volatile memory.

As many reports have pointed out, one of the potential applications of conductance quantization effect is in multi-level ultra-high-density storage [8, 170]. According to the results in “Structures, Materials, and Operation Methods of RRAM with QC Effect” section, by carefully controlling the resistive switching process of RRAM device, for example, through accurately choosing appropriate compliance current, stop voltage, sweeping speed, pulse conditions, etc., the quantized conductance values can be well controlled in theory, thus multi-level storage can be realized. Since the controllability of quantized states of CF has been confirmed to gradually improve year after year, and at the same time the kinetics and physics of switching processes and conductance quantization have also been revealed to be much clearer [16, 222], the prospect of realizing ultra-high data storage by taking advantage of the phenomenon of conductance quantization has become more promising nowadays. Except the multi-level storage, basic logic circuits can also be realized. The best achievement is from the group of Prof. M. Aono [169]. Low-power logic gates such as AND, OR, NOT gate have been configured making use of quantized conductance atomic switches (QCASs), which were fabricated by crossing metal electrode wires with solid electrolyte wires. However, in order to advance the practical multi-level high-density storage or logic circuit application of conductance quantization in RRAM, in the future, deeper investigations should be focused on how to achieve the accurate control of the quantized conductance states, and great improvements should also be required in the multi-level storage performances including endurance, retention, etc., especially based on the pulse operations.

Another important aspect related to the conductance quantization effect in RRAM devices is that the quantized CF can be made use of to investigate any other novel physical effects, such as magnetic and thermoelectric properties. Some works on the magnetic modulation in RRAM have been reported [30–42, 44–46], most of which just studied the RRAM devices with usual oxygen vacancies of CF or metal CF. Our group has also investigated the intrinsic electron transport mechanism in the formed CF by measuring the thermoelectric Seebeck effect [223]. The small-polaron hopping model can well account for the electronic transport process for all resistance states of Ti/HfO_2_/Pt device, although the corresponding resistance-temperature behaviors are contradictive. At the same time, from the point of view of device design, the controlled atomic-scale CF in simple two-terminal devices usually got at room temperature and in air can provide a media or platform to develop new one-dimensional nanodevices based on the quantum effects in CF. Here, we just show an interesting example. By replacing the electrode material with magnetic metals, we can configure the magnetic CF, and through electrical manipulation, we can try to control the CF size to get atom-sized magnetic CF. Thus, the magnetic properties such as the quantized anisotropic magnetoresistance (QAMR) effect can be studied in the atom-sized magnetic CF so as to deeply investigate the quantized transport of CF. This kind of works can provide a new characterization method for the research on the CF and the resistive switching mechanism. They might also provide a new idea of achieving stable QAMR effect in experiments and promote the deep understanding on the spin-dependent transport properties in atom-sized materials. In the long term, by simultaneously manipulating the resistance states and the ordered/disordered magnetic states, we might construct novel functional nanoscale electronic devices.

## Conclusions

In this paper, we explained the resistive switching mechanism and operating principles of filamentary RRAM and analyzed their connection with the conductance quantization effect. Then, we introduced some typical researches on the conductance quantization effect of RRAM. The device structures, switching material system, and the operating methods of RRAM related to conductance quantization effect were summarized in detail. Next, the theory and modeling of quantum transport in the atomic CF of RRAM ascribing to the conductance quantization effect were discussed. Finally, we evaluated the opportunities and challenges of the quantized CF system in RRAM devices for the multi-level storage and any other applications in the future.

## Abbreviations

2DEG: Two-dimensional electron gas
AFM: Atomic force microscopy
BD: Breakdown
CAFM: Conductive atomic force microscopy
CBRAM: Conductive bridge random access memory
CeRAM: Correlated random access memory
CF: Conductive filament
COMS: Complementary metal-oxide-semiconductor
DFT: Density-functional theory
ECM: Electrochemical metallization
emf: Electromotive force
FET: Field effect transistor
HRS: High resistance state
HRTEM: High-resolution transmission electron microscopy
IMT: Insulator-metal transition
IRS: Initial resistance state
LRS: Low resistance state
MIM: Metal-insulator-metal
MRAM: Magnetic random access memory
NVM: Non-volatile memory
PMC: Programmable metallization cell
PRAM: Phase change random access memory
QAMR: Quantized anisotropic magnetoresistance
QC: Quantized conductance
QCAS: Quantized conductance atomic switch
QPC: Quantum point contact
QW: Quantum wire
RRAM: Resistive random access memory
RS: Resistive switching
STEM: Scanning transmission electron microscopy
STM: Scanning tunneling microscope
TCM: Thermochemical mechanism
VCM: Valence change memory
Vo: Oxygen vacancy
